# Anthelmintic Activity of Ethanolic and Aqueous Extracts of *Khaya grandifoliola* Stem Bark against *Heligmosomoides polygyrus*: *In Vitro* and *In Silico* Approaches

**DOI:** 10.1155/2024/6735764

**Published:** 2024-07-17

**Authors:** Noumedem Anangmo Christelle Nadia, Mahdi Yaghoobi, Yamssi Cédric, Masoud Besati, Yungong Misparine Kiki, Ngouyamsa Nsapkain Aboubakar Sidiki, Mounvera Abdel Azizi, Vincent Khan Payne, Haibo Hu

**Affiliations:** ^1^ Department of Microbiology, Haematology and Immunology Faculty of Medicine and Pharmaceutical Sciences University of Dschang, P.O. Box 96, Dschang, Cameroon; ^2^ Laboratory of Tropical and Emerging Infectious Diseases, Buea, Cameroon; ^3^ Molecular Design and Synthesis Department of Chemistry KU Leuven, Celestijnenlaan 200F, Leuven B-3001, Belgium; ^4^ Department of Biomedical Sciences Faculty of Health Sciences University of Bamenda, P.O. Box 39, Bambili, Cameroon; ^5^ Institute for Integrative Systems Biology (I2SysBio) CSIC-University of Valencia, Paterna 46980, Spain; ^6^ Department of Medical Laboratory Sciences Faculty of Health Sciences University of Bamenda, P.O. Box 39, Bambili, Cameroon; ^7^ Department of Animal Biology Faculty of Science University of Dschang, P.O. Box 067, Dschang, Cameroon; ^8^ Jiangxi Province Key Laboratory of Pharmacology of Traditional Chinese Medicine National Engineering Research Center for Modernization of Traditional Chinese Medicine-Hakka Medical Resources Branch School of Pharmacy Gannan Medical University, Ganzhou 341000, China

## Abstract

**Background:**

Parasitic infection remains a serious health trade for humans and livestock. The purpose of this study was to present scientific proof of the anthelmintic properties of *Khaya grandifoliola*, which the native population uses to cure helminthiasis.

**Method:**

Fresh *Heligmosomoides polygyrus* eggs were isolated from faecal samples of experimentally infected mice. The faecal material was cultured, and L1 and L2 larval stages were recovered after 48 and 120 hours, respectively. Using the worm microtracker, the anthelminthic efficacy of the extracts against *H. polygyrus* was assessed. Two different extracts (aqueous and ethanol extracts) were prepared. For the ovicidal and larvicidal activities, 100 *µ*L of various concentrations of plant extracts, levamisole and 1.5% dimethyl sulfoxide (DMSO), were introduced into a 96-well microplate titer followed by the addition of 100 *µ*L of embryonated eggs (60 eggs) for the ovicidal activity and 100 *µ*L of *L*_1_ or *L*_2_ larvae (50 larvae) for the larvicidal activity. The movement of the worm was monitored for 24 hours in the worm microtracker at 27°C. The Glide module of the Schrodinger Maestro software was used to perform docking studies.

**Results:**

For the aqueous extracts, the highest percentage of inhibition of hatching was 42.77 ± 12% at 7.5 mg/mL. The IC_50_ values for the ethanol (0.36 mg/mL) extract showed that the ethanol extract had a good inhibitory effect on the ability of parasites to hatch from eggs. The inhibition percentage of L1 larvae motility at 7.5 mg/mL was 98.0 ± 1.66% and 83.33 ± 1.66% for ethanol and aqueous extracts, respectively. The negative controls, distilled water and 1.5% DMSO, had no inhibitory impact on larvae. On L1-larvae, the drug of choice levamisole (positive control) had the highest percentage effect (100.0%). Six compounds had the highest docking score and their interactions with the receptor as well. Grandiamide A interacts most with tyrosine, glycine, phenylalanine, asparagine, and serine, and its benzene ring and oxygens inhibit these receptors. Carbonyl and hydroxyl (OH) groups connect grandiamide D to asparagine, isoleucine, and phenylalanine, respectively. By donating hydrogen to the receptor through OH groups, D-glucopyranose-6-phosphate also forms relatively strong hydrogen bonds with its oxygen-bound phosphorus and the receptor. 1-O-deacetylkhayanolide E interacts most with serine and glutamic acid. The carbamic acid benzyl ester of carbamic acid [(1S)-1-phenyl-2-[(4-methylphenyl) sulfinyl] ethyl] interacts most with the receptor with carbonyl groups and with asparagine and serine. With its abundant hydroxide, D-mannitol acts as a hydrogen donor and acceptor and interacts most strongly with amino acids such as glycine, asparagine, aspartic acid, alanine, and glutamic acid.

**Conclusions:**

*K. grandifoliola* extracts possess anthelminthic properties. However, *in vivo* studies are still necessary to demonstrate the effectiveness of this plant for the treatment of helminthiasis.

## 1. Introduction

Globally, parasitic infections continue to be a serious health concern [1]. These diseases are more prevalent in tropical and subtropical regions, where the risk of infection is increased due to poor sanitary conditions and limited access to synthetic medications [1, 2]. The recent ignoring of helminthiasis in favour of the human immunodeficiency virus (HIV)/acquired immunodeficiency syndrome (AIDS), malaria, and tuberculosis as they devastate the health of nearly 1.5 billion people. This resulted in very little study being carried out on helminths; hence, they are registered as neglected tropical diseases (NTDs) [3].

Twenty-four percent (24%) of the world's population suffers from geohelminths, according to Mekonnen et al. [4]. Generally referred to as soil-transmitted helminth (STH), the parasites that cause these infections are *Ascaris lumbricoides*, *Trichuris trichiura*, and *Necator americanus*/*Ancylostoma duodenale*. More than 267 million preschool children and 568 million schoolchildren live in areas where the transmission of these parasites is intense in sub-Saharan African nations, America, China, and East Asia [5].

According to studies, gastrointestinal helminth infections affect an estimated 10 million people in Cameroon, where soil-transmitted helminths are widely dispersed throughout the nation [6]. It primarily affects school-aged children, jeopardizing their growth, intellectual development, and academic performance. These infections also increase their vulnerability to other infections such as HIV/AIDS, malaria, and tuberculosis [7]. Common symptoms associated with parasitic helminthiasis include malnutrition, anaemia, diarrhoea, asthenia, lethargy, and anorexia that compromise human and animal health [8].

Patients often employ synthetic anthelmintic medications to control these parasitic diseases. But, regrettably, the poor usage of synthetic drugs and the nonrespect doses have led to the development of resistance [9]. Furthermore, some of the synthetic drugs have side effects [10]. Medicinal plants remain one of the most reliable solutions to address the issue of synthetic drug resistance, adverse effects, and high cost. Furthermore, they are less toxic to the environment since they exist naturally in the environment [11]. However, indigens have little or no knowledge about the use of these plants, and when they do, the spectrum or the extent to which such plant products can act is unknown. This limits the exploitation of the therapeutic virtues of these plants. Therefore, a systematic screening of their anthelminthic activity against a wide range of helminths may be of great significance to the local population as well as to the pharmaceutical industry. It is in this regard that we focused our research towards the search for plants endowed with pharmacological properties against disease-causing helminths. Since ancient times, herbal treatment has been used to limit the emergence of parasite resistance [12]. According to some researchers, phytochemicals that have anthelmintic activity, such as glycosides, alkaloids, tannins, terpenoids, and flavonoids, are important [13, 14].

*Khaya grandifoliola*, also called African mahogany, is widely used in African folk medicine due to its pharmacological relevance. Traditional healers in Cameroon treat helminthiasis through the use of plants. Guy-Armand et al. [15] demonstrated antiplasmodial and cytotoxicity activity of this plant. There was another discovery that stem bark had antiulcer properties, antianaemic, hypoglycaemic, and hypoproteinaemic effects [16]. *K. grandifoliola* has a gum that is used as a tablet binder for paracetamol [17]. Several extracts are safe at different therapeutic dosages, according to toxicity testing [18].

Traditionally, *in vitro* screening methods for parasitic worms have been limited by their lack of computerization and reproducibility [19]. The worm *Heligmosomoides polygyrus* is a parasite that is similar in all aspects to human nematodes and hence is used as a model for anthelminthic drug discovery [20]. The purpose of this study was to provide scientific evidence on the anthelmintic activities of *K. grandifoliola* used by the local community as a helminthiasis therapy.

## 2. Materials and Methods

### 2.1. Helminth Parasite

Pr. Rick Maizels of the University of Edinburgh in the United Kingdom kindly provided the infectious third-stage larvae (L3) of *H. polygyrus. H. polygyrus* is a parasitic nematode that serves as a mouse model for the assessment of anthelminthic medications and replicates the infection caused by strongylid nematodes [20, 21].

### 2.2. Collection and Identification of Plant Species

*K. grandifoliola's* leaves, flowers, and fruits were collected in the western region of Cameroon in the dry season of December 2023 and recognized by the National Herbarium using the voucher specimen number 52658/HNC.

### 2.3. Preparation of Extracts

Because traditional healers during the survey told us that they use fermented palm wine (ethanol) or infusion for the preparation of this medicine, we decided to prepare ethanol and aqueous extracts. One kilogram of the fresh sample of the stem back was collected. After drying and grinding the stem bark, we had 670 g of powder. The aqueous and ethanol extracts were made using the procedure outlined by Wabo et al. [22]. In brief, one litter of 95% ethanol was mixed with 100 g of plant powder and homogenized. The mixture was mixed daily for 72 hours. Whatman paper number 1 and cotton were used to filter the homogenate. The filtrate was dried in an oven at 40°C to remove the ethanol. For the aqueous extract, 100 grams of powder were mixed with one litter of warm distilled water and the mixture was then allowed to cool before being filtered through cotton and Whatman paper number 1. After that, the filtrate (100 ml) was poured into a steel tray and dried in a convection oven at 45°C until a constant dry mass (dried aqueous extract) was obtained [23].

### 2.4. Collecting and Concentrating *H. polygyrus*-Embryonated Eggs

The flotation technique was used for collecting the eggs from faecal samples containing *H. polygyrus* [24]. In brief, the eggs obtained from the flotation technique were centrifuged at 1500 rpm for 10 min after being rinsed with distilled water to wash the salt from the egg. After a series of washings of the eggs, which was meant to extract the salt solution, the salt-free eggs were then incubated for 24 hours to obtain embryonated eggs for the assay.

### 2.5. Coproculture and Retrieval of L1 and L2 Larvae of *H. polygyrus*

Embryonated eggs were then incubated at 25°C for 24 and 72 hours in order to produce L1 and L2 larvae, respectively, according to the method described by Cédric et al. [20]. The larvae were distinguished according to their shape.

### 2.6. Preparation of Different Concentrations of Extracts

A stock solution of concentration 15 mg/mL was prepared by weighing 0.15 g of extract using an electronic balance and mixed with 10 mL of distilled water in a 50 mL beaker.

Another stock solution of a concentration of 10 mg/mL was obtained from the 15 mg/mL stock solution. This mixture was homogenized using a vortex mixer and then sonicated (to facilitate the dissociation of the extract). By successive dilutions, we obtained solutions with concentrations of 0.625 mg/mL, 1.25 g/mL, 2.5 mg/mL, 5.0 mg/mL, and 7.5 mg/mL. Dimethyl sulfoxide (DMSO), which aids in dissolving the ethanol extract, was combined with the same amount of dry extract for the ethanol extract to obtain the same concentrations.

### 2.7. Egg-Hatching Test

The approach outlined by Cédric et al. [20, 24] was used to assess the extract's anthelminthic efficacy. In 96-well round bottom microtiter plates, 100 *µ*L of 60 embryonated eggs were put in contact with 100 *µ*L of the extract at various concentrations (15, 10, 5, 2.5, and 1.25 mg/mL) to obtain final concentrations ranging from 7.5 to 0.625 mg/mL in a final volume of 200 *µ*L. Five molar (5 M) levamisole and 1.5% dimethyl sulfoxide (DMSO) were used as positive and negative controls, respectively. The plate was subsequently incubated in the worm microtracker, a device that monitors worm movement. Once hatching occurred, the continuous movements of larvae in each well were recorded by the worm microtracker. Using the following formula, the percentage of inhibition of egg-hatching was determined [25]:

(1) $$\begin{array}{c} \%\text{ inhibition}=\frac{\text{mobility activity of negative control}-\text{mobility activity of the test sample}}{\text{mobility activity of negative control}}\times 100. \end{array}$$

### 2.8. Larval Motility Assays

The larvicidal activity of the extract against *L*_1_ and *L*_2_ was evaluated using the approach outlined by Cédric et al. [20, 24]. One hundred microliters of 50 L1 larvae were put in contact with the extract at various concentrations (7.5 to 0.3125 mg/mL) in 96-well round bottom microtiter plates. Levamisole and 1.5% DMSO were used as positive and negative controls, respectively.

The worm microtracker, a device that measures worm motility, was then used to incubate the 96-well microtiter plate. The percentage of inhibition of larvae motility was determined [26]. The same procedure was used to evaluate the effects of the extracts on L2 larvae.

### 2.9. Phytochemical Screening

The screening of the aqueous and ethanol extracts of *K. grandifoliola* for phenol and flavonoids, as well as for sterols, alkaloids, triterpenoids, saponins, anthocyanins, and anthraquinones was performed quantitatively and qualitatively, as previously reported by Guy-Armand et al. [15].

### 2.10. Preparation of the Ligand

Using Maestro, which was designed to supply input structures for the Glide and PHASE modules, the ligand was prepared using the Ligand Preparation [27] module. With the help of special algorithms, Clean Up Wizard can handle one ligand per second at a time, efficiently transforming massive datasets from 2D to 3D structures and crucial docking study processes.

### 2.11. Molecular Docking

**Figure 1:**
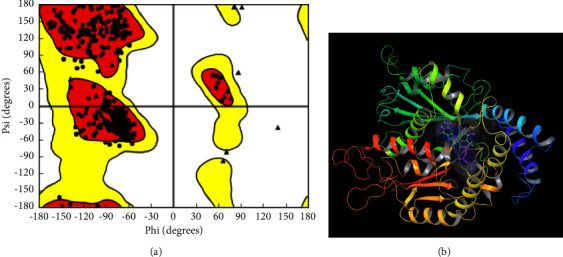
(a) Ramachandran plot of the tubulin alpha-1B chain and (b) optimized tubulin alpha-1B chain with the optimized active site.

Based on information from several publications, the anthelmintic receptor protein [28, 29] as shown in Figures 1(a) and 1(b), the tubulin alpha-1B chain was selected as the traditional target for a variety of anthelmintic drugs in this investigation. The structure of *Caenorhabditis elegans* tubulin was downloaded from the Protein Data Bank (PDB ID: 6E88) portal. Hydrogen bonds matching pH 7.4 were added after crystallographic water molecules, or those without 3H bonds were eliminated, taking into account the proper ionization states for basic and acid amino acid residues. The crystal structure's energy was minimized using the OPLS_2005 force field [30]. Approximately 117 *K. grandifoliola* ligands were used to construct the coupling, and the location of this protein was identified using the programs Glide and Sitemap [31].

The tubulin alpha-1B chain's grid box generation was handled using Maestro's Glide software, specifically the receptor grid generation part. We take advantage of Maestro's Sitemap program since it can anticipate the receptor's active location. Ultimately, for the active site of the tubulin alpha-1B chain, the grid center was calculated using the dimensions (A) of the inner box, which measured 15 × 15 × 15, and the outer box, which measured 20 × 20 × 20 (*X*: 240.06, *Y*: 91.29, *Z*: 118.29).

The Glide module of the Schrodinger Maestro software was used to conduct the docking studies [27]. The software score function was used to classify and rank several potential adduct structures produced by molecular docking [32]. It makes predictions about the three-dimensional structure of any complex based on the binding characteristics of the ligand and target. Docking is the process of predicting the direction and conformation of a ligand within a particular binding site. The “protein preparation wizard” in Maestro Wizard was used to preprocess the protein structure. By autonomously constructing the module's state and refinement step phases, hydrogen atoms and a few necessary bonds were added to the missing protein molecule. Following the optimization procedure, the construction of receptor grids was handled, and the docking scores were examined using various docked ligand conformations [33, 34].

### 2.12. Statistical Analysis

The results were analysed using GraphPad Prism version 8.0, a statistical tool. The IC_50_ was determined using the concentration-response curves that were produced by plotting the logarithm of the concentration as a function of the percentage inhibition. It is important to use the IC_50_ value because it indicates how much a drug is needed to inhibit a biological process by half. The Glide module of the Schrodinger Maestro software was used to perform docking studies. The software's score function was used to classify and rank various potential adduct structures produced by molecular docking.

## 3. Results

### 3.1. Anthelminthic Test

The effect of ethanol and aqueous extracts of *K. grandifoliola* on the inhibitory percentage (%), hatching, and inhibitory concentration (IC_50_) of larvae motility is shown in Table 1.

Aqueous extracts had the highest percentage of hatching inhibition (42.77 ± 12%) at the concentration of 7.5 mg/mL. While at 0.625 mg/mL concentration, the lowest percentage of hatching inhibition of 6.77 ± 3.33% and 10.0 ± 2.89% was obtained for ethanol and aqueous extracts, respectively. A 100% hatching in distilled water and 1.5% DMSO suggests that the plant extract affected the worm. As the concentration increased, the percentage of inhibition of egg-hatching increased correspondingly.

The IC_50_ value for the ethanol (0.36 mg/mL) extract showed that the ethanol extract had a strong inhibitory effect on the ability of the parasites to hatch from the eggs. Based on the analysis of this table, the concentration of 7.5 mg/mL resulted in the highest percentage of inhibition in L1 larvae, 98.0 ± 1.66% for the ethanol extract, and 83.33 ± 1.66% for the aqueous extract, respectively.

At 0.625 mg/mL, the lowest percentage of inhibition was observed, with the ethanol and aqueous extract showing 75 ± 8.66% and 21.67 ± 10.93% inhibition, respectively. At the maximum degree of inhibition, there was no significant difference in their levels of inhibition; however, at the lowest percentage of inhibition, there was a significant difference. As the concentration increases, so does the percentage of inhibition.

As seen by the mean inhibition percentages of 0% for both distilled water and 1.5% dimethyl sulfoxide (negative control), the negative controls did not show any inhibitory effect on the larvae. The medication of preference (positive control), levamisole, had the highest proportion (100.0%) of L1-larvae. According to this value, levamisole inhibits the development of *H. polygyrus* L1 larvae. The IC_50_ values for ethanol and aqueous extracts for L1 larvae were 9.938 mg/mL and 0.0001467 mg/mL, respectively, according to this table. This clearly demonstrated that the ethanol extract was less effective than the aqueous extract. With a significant difference (*p* < 0.0001), the concentration of 7 mg/mL of ethanol and aqueous extracts showed the highest mean inhibition rates in L2 larvae, at 98.32.9% and 83.32.9%, respectively. Levamisole showed 100% inhibition, while distilled water and 1.5% dimethyl sulfoxide (negative control) did not impact. In L2 larvae, the aqueous and ethanol extracts showed inhibitory percentages of 21.67 ± 18.52% and 75 ± 15%, respectively, at the lowest dose of 0.625 mg/mL. There was a significant difference in terms of their inhibition at both the highest and lowest levels of inhibition. The percentage of inhibition increases with increasing concentration. With an IC_50_ for L2 larvae of 0.1937 mg/mL while that of the aqueous extract was undetermined.

### 3.2. The Phytochemical Screening

Similar to the ethanol extract, saponins are absent, but all other constituents are present. The aqueous extract had 162.2 ± 48.20 mg/g of flavonoids, and the ethanol extract had a flavonoid concentration of 448.9 68.85 mg/g. Similar to this, the ethanol extract contained more phenolic chemicals (631.916.44 mg/g) than the aqueous extract (372.47.328 mg/g).

### 3.3. Anthelminthic Molecular Docking Analysis

Molecular docking between components (ligands) and the target protein was carried out using the Glide module [35, 36]. The docking scores of the tubulin alpha-1B chain and ligands are displayed in Table 2. On the basis of the examination of this table, it may be inferred that certain ligands exhibited elevated docking scores during their interactions with the target protein's amino acids. As can be observed in Figure 1, which illustrates the impact of this molecule's hydrophobic chain, the purple arrows represent the negative bonds of the ligands, while the green arrows represent hydrophobic interactions [37, 38].

HTVS, SP, and XP molecular docking techniques were utilized to screen the chemicals that were isolated from *K. grandifoliola.* 15% of the most stable ligands with docking scores were examined in each phase. The most stable ligand structures were selected using the XP docking score approach.

**Figure 2:**
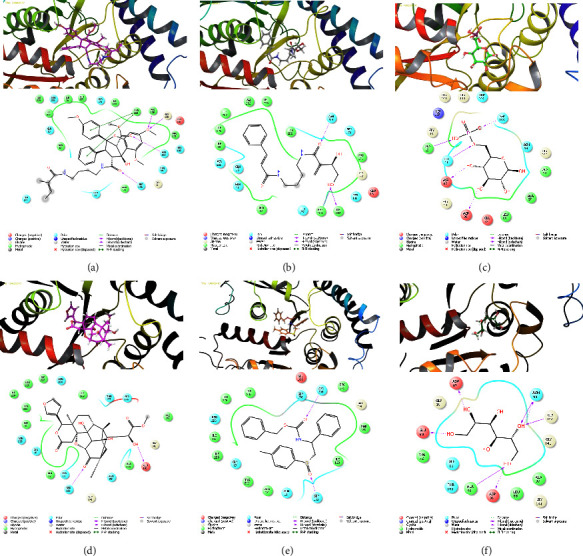
3D and 2D interactions of receptor protein and *K. grandifoliola* ligands: (a) grandiamide A, (b) grandiamide D, (c) D-glucopyranose-6-P, (d) 1-O-deacetylkhayanolide E, (e) [(1S)-1-phenyl-2-[(4-methylphenyl) sulfinyl] ethyl] carbamic acid benzyl ester, and (f) D-mannitol.

Figure 2 shows the six compounds that had the highest docking score and their interactions with the receptor as well. Grandiamide A interacts most with tyrosine, glycine, phenylalanine, asparagine, and serine, and its benzene ring and oxygens inhibit these receptors. Carbonyl and hydroxyl groups connect grandiamide D to asparagine, ile, and phenylalanine, respectively. By donating hydrogen to the receptor through OH groups, D-glucopyranose-6-P also forms relatively strong hydrogen bonds with its oxygen-bound phosphorus and the receptor. 1-O-Deacetylkhayanolide E interacts most with serine and glutamic acid. The carbamic acid benzyl ester of carbamic acid [(1S)-1-phenyl-2-[(4-methylphenyl) sulfinyl] ethyl] interacts most with the receptor with carbonyl groups and with asparagine and serine. With its abundant OH, D-mannitol acts as a hydrogen donor and acceptor and interacts most strongly with amino acids such as glycine, asparagine, aspartic acid, alanine, and glutamic acid.

## 4. Discussion

The egg-hatching mechanism was not highly suppressed by both the aqueous and ethanolic extracts of *K. grandifoliola*, and the ethanol extract had the strongest effects (IC_50_ = 0.36 mg/mL). It is possible that the inability of the *K. grandifoliola* extract to suppress the events leading up to hatching is the cause of these low ovicidal inhibition percentages. The permeability properties of the eggshell and the concentration of trehalose in the perivitelline fluid are two examples of parameters that affect hatching and are crucial for the survival of the egg, unhatched juveniles [39]. Our extracts were unable to affect any of these indicators.

These findings are in contradiction to those of Zangueu et al. [40], who found that extracts of *Maytenus senegalensis* extracts inhibited egg-hatching, leading to an ovicidal effect with a significant (*p* < 0.01), concentration-dependent suppression of egg-hatching (*p* < 0.01). The 100% hatching in the presence of distilled water and 1.5% DMSO suggest that they have no influence on hatching. Ngouateu Teufack et al. [41] reported similar results. According to Ngouateu Teufack et al. [41], dimethyl sulfoxide and Tween 80 are frequently used as vehicles in the anthelminthic screening of medicinal plants because they do not have any effect on egg-hatching and larvae at concentrations less than 10%. Subedi [42] suggests that the tannins in *K. senegalensis* extract may be the cause of the anthelmintic activities observed *in vitro.* According to Greiffer et al. [43], tannin binding caused an increase in the stiffness of the worms' cuticle. This could be a crucial discovery to explain a number of anthelmintic behaviours linked to tannins, including inhibition of motility and molting or exsheathing.

When investigated *in vitro*, the anthelmintic activity of two commercial products containing tannins and the common forage plant sainfoin both demonstrated anthelmintic activities [44]. The presence of secondary metabolites such as saponins is shown by phytochemical screening and does not necessarily indicate an anthelminthic activity because not all saponins have anthelminthic activities [45]. The saponins aescin and digitonin, as shown by Santos et al. [45], have a strong *in vitro* anthelmintic action, and the glycone component of both saponins is crucial to this activity.

The development of *H. polygyrus* hatching structures, such as the protractible stylet, which is required to open the eggshell during hatching, may not have been suppressed by plant extracts explaining this poor hatching activity. An earlier study by Cédric et al. [24] found that the ethanol extract was more active than the aqueous. Both ethanol and water are polar solvents that can be used to extract different polar compounds present in plants. It is possible to extract components that are soluble in water and oil using ethanol. This could be explained by the superior ovicidal activity of the ethanol extract over that of the aqueous.

The mean larval motility rate decreases as the concentration increases from 0.625 mg/mL to 7.5 mg/mL. These findings are comparable to those of Kolapo et al. [46], who found that the ethyl acetate fraction (80 mg/mL + Tween 80) and the crude extract of *K. grandifoliola* at 200 mg/mL and 400 mg/mL both had 100% anthelmintic activity compared to the standard (5 mg of levamisole).

Throughout this investigation, we discovered that L2 larvae were more sensitive to extracts than L1 larvae. These results were consistent with past Ngouateu reports [41]. These scientists claim that because the L1 stages have just recently hatched, they still have some food or energy reserved. As time passes, this food and energy are depleted, increasing the sensitivity of the stages to the substances under study.

In the late eluting fractions of the methanolic extract of *K. senegalensis* leaves, Subedi [42] found three limonoids: mahonin (1), methyl angolensate (2), and a new molecule called 16-oxodelevoyin B (3). Mahonin (1) was the most active of the three limonoids, all of which showed anthelmintic bioactivity. The extracts may have a larvicidal effect because they diffuse through the cuticle of the larvae. The findings of da Rocha et al. [47] are supported by this outcome.

According to Moussouni, et al. [48], the paralysation observed in the larvae is due to the blockage of receptors by the extract at the postsynaptic membrane level, therefore inhibiting the transmission of nerve impulses. The larvicidal activity of the extracts may also be due to compounds such as tannins, which interact with membrane-associated sterols at the level of the cuticle, disrupting cell permeability [49].

Similar to our findings, in a study conducted by China et al. on larval migration and adult motility, *K. senegalensis* methanolic extract had the highest level of efficacy [50]. These authors state that the lethal effect was dose-dependent and that, when exposed to 2400 *μ*g/mL of the acetonic extract or 1200 *μ*g/ml of the methanolic extract of *K. senegalensis*, respectively, 75% and 100% of the worms were deactivated within 24 hours. Secondary metabolites are found by phytochemical screening and may be the cause of the ovicidal and larvicidal effects. Other researchers, such as [24, 51, 52], have shown that certain phytochemical components, such as carotenoids, triterpenes, saponins, steroids, coumarins, tannins, glycosides, enzymes, anthraquinones, essential oils, lipids, protein, and fibers, must be present for a plant to have the anthelminthic activity.

Considering the antihelminthic effect of *K. grandifoliola* plant extract and its evidence in a recent article, its molecular docking studies, which have been investigated regarding the inhibitory effect on tubulin alpha-1B chain protein and ligand by ligand, show that the effect can be greater due to the presence of compounds such as grandiamide A, grandiamide D, D-glucopyranose-6-P, 1-O-deacetylkhayanolide E, as well as [(1S)-1-phenyl-2-[(4-methylphenyl) sulfinyl [ethyl] carbaci acid benzyl ester. As a result, we can conclude that the compounds in *K. grandifoliola* have better antihelminthic properties than other compounds on the basis of the results of the molecular docking studies.

## 5. Conclusions

The present study showed that L2 larvae were more sensitive to extracts than L1 larvae and the extract had good larvicidal activities. Furthermore, *K. grandifoliolas* have several compounds such as grandiamide A, grandiamide D, D-glucopyranose-6-P, 1-O-deacetylkhayanolide E, as well as [(1S)-1-phenyl-2-[(4-methylphenyl) sulfinyl [ethyl] carboxylic acid benzyl ester which presented a very powerful anthelmintic activity docking score. None of these compounds had been seriously considered important in the field of parasitology. This suggests that these extracts can be used to treat helminthiasis.

### 5.1. Limitation of the Present Findings

To scientifically support the use of *K. grandifoliola* in Cameroonian folk medicine in the fight against helminths *in vivo*, toxicity research is necessary.

## Figures and Tables

**Table 1:** Effect of ethanol and aqueous extracts of *K. grandifoliola* on the inhibitory percentage (%), hatching, and inhibitory concentration (IC_50_) of larvae motility.

Anthelminthic test	Extract	Concentrations (mg/mL)						Controls		
		0.625	1.25	2.5	5	7.5	IC_50_	5 M levamisole	1.5% DMSO	0.9% NaCl
% inhibition of hatching	Aqueous	10 ± 2.88^a^	17.33 ± 1.66^a^	20 ± 0.0^a^	45 ± 8.66^b^	42.77 ± 12^b^	Undetermined	100.00 ± 0^c^	0.00 ± 0^a^	0.00 ± 0^a^
	Ethanol	6.77 ± 3.33^a^	12.77 ± 4.4^a^	20 ± 2.88^a^	19.4 ± 1.6^a^	21.77 ± 6^b^	0.36	100.00 ± 0^c^	0.00 ± 0^a^	0.00 ± 0^a^

% inhibition of L1 larvae motility	Aqueous	36.67 ± 6^b^	71.61 ± 1.67^b^	88.33 ± 4.4^b^	91.67 ± 1.67^b^	95 ± 2.8^b^	0.00014	100 ± 0^b^	0.00 ± 0^a^	0.00 ± 0^a^
	Ethanol	58.33 ± 1.67^b^	65 ± 2.87^b^	71.67 ± 1.66^b^	81.67 ± 4.4^b^	90 ± 5.77^b^	9.94	100 ± 0^b^	0.00 ± 0^a^	0.00 ± 0^a^

% inhibition of L2 larvae motility	Aqueous	21.67 ± 10.93^a^	25 ± 8.66^b^	43.33 ± 3.33^b^	53.33 ± 3.33^b^	83.33 ± 1.67^b^	Undertermined	100 ± 0^b^	0.00 ± 0^a^	0.00 ± 0a
	Ethanol	75 ± 8.66^b^	85 ± 7.6^b^	98.33 ± 1.66^b^	100 ± 0.0^b^	98.33 ± 1.68^b^	0.19	100 ± 0^b^	0.00 ± 0^a^	0.00 ± 0^a^

a, b, c,…: for the same row and different concentrations, the values that carry the same superscript letter are not significantly different from the negative control at *p* < 0.05. DMSO: dimethyl sulfoxide.

**Table 2:** Docking scores of the ligands and the tubulin alpha-1B chain.

No.	Compounds	Docking score
1	Grandiamide A	−8.591
2	Grandiamide D	−7.845
3	D-Glucopyranose-6-P	−7.566
4	1-O-Deacetylkhayanolide E	−7.435
5	[(1S)-1-Phenyl-2-[(4-methylphenyl) sulfinyl] ethyl] carbamic acid benzyl ester	−7.108
6	D-Mannitol	−7.006
7	Khayalactone	−6.796
8	D-Glucopyranose-6-P	−6.564
9	Cholestan-3,26-diol-22-one	−6.47
10	Khayasin	−6.391
11	6-Hydroxyflavanone	−6.382
12	Grandiamide B	−6.323
13	Alpha-11-selinene-4-ol	−5.929
14	Terpinylacetate	−5.822
15	Cedrelanol	−5.801
16	7-Deacetylkhivorin	−5.675
17	Cholestan-3,26-diol-22-one	−5.661
18	Methyl behenate	−5.545
19	Alpha-muurol-5-En-4-Ol	−5.542
20	4-Carvomenthenol	−5.409

## Data Availability

All data generated and analysed are included within this research article.

## Abbreviations

AIDS: Acquired immunodeficiency syndrome
DMSO: Dimethyl sulfoxide
HIV: Human immunodeficiency virus
NTD: Neglected tropical diseases
OH: Hydroxyl.
